# Botulinum Toxin Type A in Red Ear Syndrome and a Novel Multi-nerve Injection Paradigm: A Case Report

**DOI:** 10.7759/cureus.96617

**Published:** 2025-11-11

**Authors:** Mya T Clark, Kimberley E Meathrel

**Affiliations:** 1 Research, Bespoke Skin MD, Kingston, CAN; 2 Plastic Surgery, Bespoke Skin MD, Kingston, CAN

**Keywords:** auricular erythema, botulinum injection, botulinum toxin type a, neurovascular alterations, • red ear syndrome

## Abstract

Red ear syndrome (RES) is a rare, poorly understood disorder characterized by episodic ear erythema and pain. Its pathophysiology remains unclear, with proposed associations to migraine, erythromelalgia, and rosacea. Existing treatments are often ineffective, emphasizing the need for novel approaches. We describe a 33-year-old male with bilateral, non-headache RES refractory to standard therapies. A novel botulinum toxin type A (BoNT-A) injection paradigm, targeting both sensory and autonomic innervation, was implemented. Although episode frequency remained unchanged, the patient experienced significant reductions in pain intensity, episode duration, and improvement in quality of life.

## Introduction

Red ear syndrome (RES) is a rare condition characterized by episodic pain and erythema of one or both ears [1]. First described and coined by Lance, it remains a diagnosis of exclusion due to its unclear pathophysiology and variable response to treatment [2,3].

Proposed mechanisms include dysfunction of trigeminal-autonomic pathways (central) and dysfunction of upper cervical nerves, particularly the C3 root (peripheral) [1]. Current management often borrows from migraine therapy with inconsistent efficacy, complicating standardized care [4-6].

Botulinum toxin type A (BoNT-A) has emerged as a promising therapeutic option with case reports showing positive outcomes [7-9]. Proposed mechanisms include inhibition of neurotransmitter release, modulation of nociceptive signaling, and reduction of parasympathetic outflow [10,11]. Furthermore, RES may share pathophysiological overlap with vascular pain syndromes, erythromelalgia (EM), and rosacea [12-15].

## Case presentation

A 33-year-old Caucasian male presented to the clinic with bilateral primary RES first noted in 2018. He reported episodes of erythema and intense heat of both ears, describing the pain as sharp or shooting along the auricular rim.

The patient experienced three to four daily flare-ups, shorter during the day but often prolonged in the evening, disturbing sleep. Triggers included mechanical stimuli (e.g., hair brushing, headphones, lying on the ear), heat, sun exposure, and exertion. 

The patient reported that extensive past investigations, including multiple MRIs, endocrinology workup, and rheumatologic workup, were unremarkable. Relapsing polychondritis was ruled out, and RES was diagnosed. Original test data were not accessible for independent verification. Trialed pharmacologic therapies (topiramate, naproxen, indomethacin, colchicine, celecoxib, and topical anesthetics) were largely ineffective, with some poorly tolerated.

By 2023, while continuing topiramate and gabapentin, the patient consented to off-label BoNT-A (Jeuveau, prabotulinumtoxinA) injections. A total of 20 units per ear were administered anteriorly and posteriorly every three to four months.

**Figure 1:**
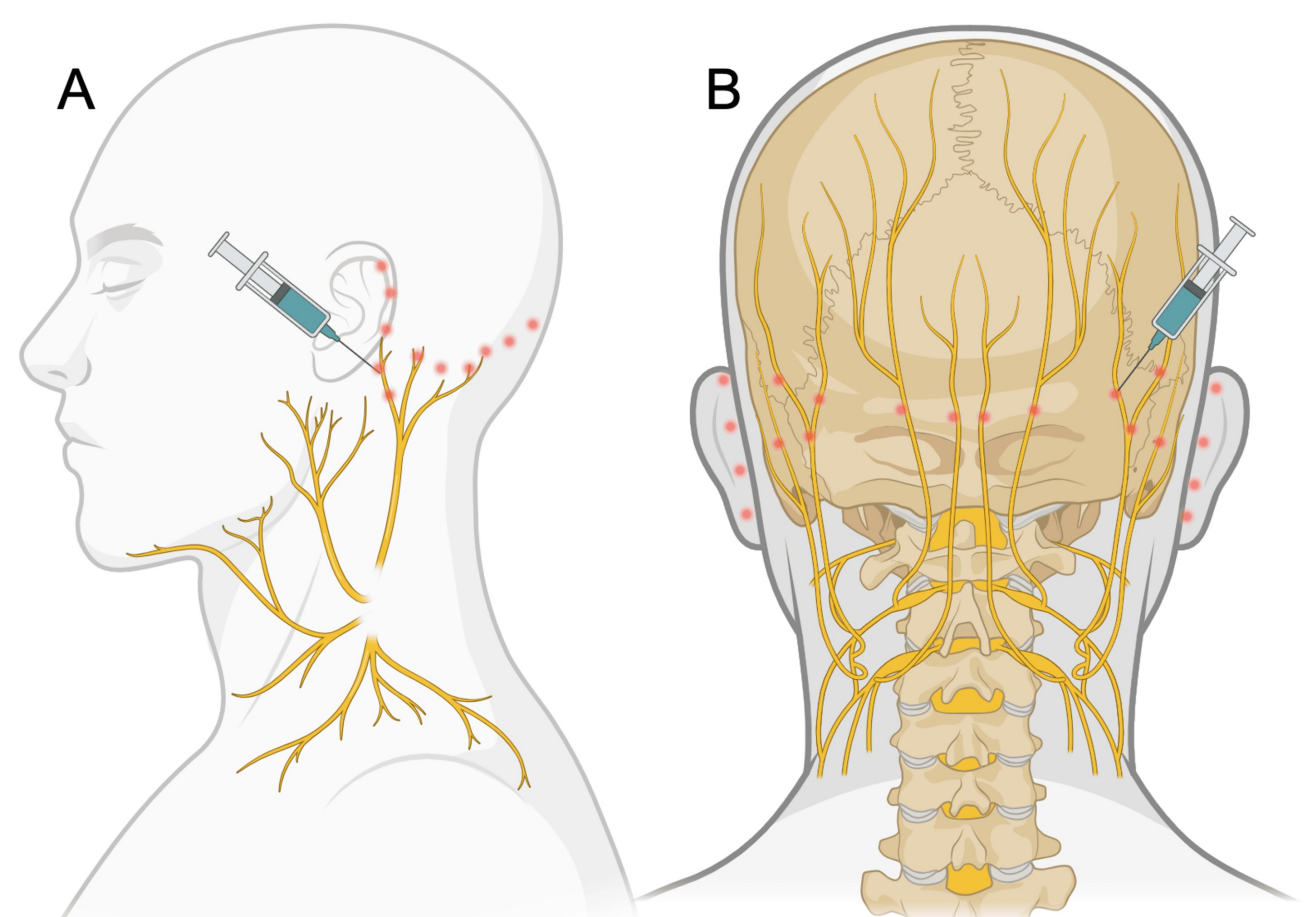
Multi-nerve botulinum toxin type A injection paradigm for red ear syndrome (A) Lateral view showing injection sites targeting the greater auricular nerve, lesser occipital nerve, and posterior auricular branch. (B) Posterior view illustrating additional injection sites targeting the greater occipital nerve and posterior auricular region. Created in BioRender by Meathrel (2025) https://BioRender.com/rfq91w3.

Initial injections were administered subcutaneously along the pinna as described by Boulton [9]. However, this provided no relief. Subsequent sessions employed an anatomically informed, multi-nerve approach targeting both sensory and autonomic nerve supply, including the greater occipital nerve, the lesser occipital nerve, the greater auricular nerve (along its anatomical path), and the posterior auricular groove targeting the posterior auricular branch of the facial nerve (Figures 1A-1B). This injection paradigm for RES treatment has not been previously described.

Following this approach, flares became milder, shorter in duration (<1 hour from three to four hours), and easier to manage. The patient described BoNT-A as “life-changing” and has maintained this injection paradigm to the present date.

## Discussion

Rationale for a multi-nerve injection paradigm 

Our patient’s presentation and initial response mirrored a previously reported unsuccessful case. Although Boulton et al. did not specify the rationale or neural targets, their method also provided minimal relief in a patient with secondary RES [9].

By targeting multiple neural pathways (peripheral, spinal, and cranial), our novel approach aimed to modulate both nociceptive signaling and autonomic dysregulation, processes implicated in RES [4]. 

Prior studies by Tolebeyan and Castellanos-Gonzalez et al. reported symptomatic improvements with BoNT-A but provided limited detail on injection patterns or dosing [7,8]. Our case contributes anatomical specificity within the context of this therapeutic approach.

Red ear syndrome subtypes 

Our observations illustrate variability in RES presentation. Tolebeyan described a patient whose headache frequency decreased by approximately 75%, whereas our patient did not experience a decrease in episode frequency, possibly due to trigger dependence or budget-limited dosing. Tolebeyan noted that the erythema was unrelated to their headaches, though this remained unexplained [7]. Castellanos-Gonzalez et al. observed improvement lasting up to five months before symptom recurrence [8]. Our patient’s results were similar but relapsed sooner (~4 months).

Unlike prior reports associating RES with headache or trigeminal-autonomic features [16,17], our patient did not report headache symptoms and exhibited strong environmental triggers. These findings are consistent with an EM-like presentation, characterized by heat-triggered erythema and burning pain relieved by cooling [12,17-19].

Features observed in our patient, such as persistent erythema and residual flares despite nerve-targeted BoNT-A, resemble patterns described in EM and rosacea [15]. BoNT-A has been reported to improve neurovascular features in rosacea, which may provide context for its use in this case [20].

## Conclusions

Observations from this case demonstrate that a multi-nerve BoNT-A injection paradigm can reduce the severity and duration of refractory RES flares. Compared to a superficial approach, personalized targeting of neural pathways provided meaningful relief, including patients with EM-like features. This approach may offer a practical option for improving the quality of life in patients with resistant RES.
